# Characterization and Expression Analysis of *PtAGL24*, a *SHORT VEGETATIVE PHASE*/*AGAMOUS-LIKE 24 (SVP/AGL24*)-Type MADS-Box Gene from Trifoliate Orange (*Poncirus trifoliata L.* Raf.)

**DOI:** 10.3389/fpls.2016.00823

**Published:** 2016-06-10

**Authors:** Lei-Ming Sun, Jin-Zhi Zhang, Chun-Gen Hu

**Affiliations:** Key Laboratory of Horticultural Plant Biology, College of Horticulture and Forestry Science, Huazhong Agricultural UniversityWuhan, China

**Keywords:** flowering, floral development, MADS-box, trifoliate orange, *PtAGL24*

## Abstract

The transition from vegetative to reproductive growth in perennial woody plants does not occur until after several years of repeated seasonal changes and alternative growth. To better understand the molecular basis of flowering regulation in citrus, a MADS-box gene was isolated from trifoliate orange (precocious trifoliate orange, *Poncirus trifoliata* L. Raf.). Sequence alignment and phylogenetic analysis showed that the MADS-box gene is more closely related to the homologs of the *AGAMOUS-LIKE 24* (*AGL24*) lineage than to any of the other MADS-box lineages known from *Arabidopsis*; it is named *PtAGL24*. Expression analysis indicated that *PtAGL24* was widely expressed in the most organs of trifoliate orange, with the higher expression in mature flowers discovered by real-time PCR. Ectopic expression of *PtAGL24* in wild-type *Arabidopsis* promoted early flowering and caused morphological changes in class I transgenic *Arabidopsis*. Yeast two-hybrid assay revealed that PtAGL24 interacted with *Arabidopsis* AtAGL24 and other partners of AtAGL24, suggesting that the abnormal morphology of *PtAGL24* overexpression in transgenic *Arabidopsis* was likely due to the inappropriate interactions between exogenous and endogenous proteins. Also, PtAGL24 interacted with SUPPRESSOR OF OVEREXPRESSION OF CONSTANS1 (PtSOC1) and APETALA1 (PtAP1) of citrus. These results suggest that *PtAGL24* may play an important role in the process of floral transition but may have diverse functions in citrus development.

## Introduction

The optimal timing of transition from vegetative to reproductive growth, known as the floral transition, is crucial for successful sexual reproduction of flowering plants. This developmental transition is precisely regulated by various environmental stimuli and endogenous signals, such as light, temperature, nutrients, and plant age (Wellmer and Riechmann, 2010; Khan et al., 2014). In recent decades, based on intensive studies of genetic and molecular mechanisms of *Arabidopsis*, an intricate regulatory network of several major genetic pathways that control the floral transition has been revealed (Boss et al., 2004; Wang J.-W. et al., 2009; Khan et al., 2014). The photoperiod and vernalization pathways respond to environmental cues. The autonomous and age pathways regulate flowering by monitoring specific developmental states of plants, whereas the gibberellin pathway particularly mediates flowering in non-inductive, short-day conditions (Wang J.-W. et al., 2009; Wang R. et al., 2009; Khan et al., 2014). The interaction among these signaling pathways regulates a group of common targets, the floral pathway integrators, including *FLOWERING LOCUS T* (*FT*), *SUPPRESSOR OF OVEREXPRESSION OF CONSTANS 1* (*SOC1*), *FLOWERING LOCUS* (*FLC*), and *LEAFY* (*LFY*) (Moon et al., 2003; Wigge et al., 2005). These genes are also key regulators of flowering time and could regulate the transition from the juvenile phase to the adult phase in woody plants (Khan et al., 2014). However, the underlying molecular mechanism of flowering time may differ between perennial plants and *Arabidopsis* because of different flowering characteristics, such as the long juvenile phase and seasonal flowering. Therefore, an understanding of these different characteristics requires identification and characterization of flowering genes related to these characteristics in woody plants.

Citrus, an evergreen fruit tree of *Rutaceae*, is one of the most important and widely grown fruit crops in the world. The commercial value of citrus is mainly focused on the fruits, which can be consumed fresh or produced for juice, jam, and wines (Tan and Swain, 2007). Flowering is an essential step for fruit trees and significantly affects the economic benefit of fruit production. For citrus, it often takes 6–20 years for flowering to occur after seed germination (Pena et al., 2001). This long juvenile phase makes the traditional breeding approaches too time consuming to meet the increasing market demand. Therefore, elucidation of the molecular mechanism of flowering in citrus plants is important for accelerating floral transition by genetic engineering. In *Arabidopsis*, the floral transition and floral organ identity are controlled by a subset of MADS-box transcription factors such as *AGL24* and *SHORT VEGETATIVE PHASE* (*SVP*). These two closely related MADS-box genes have been shown to be important for various stages of reproductive development (Michaels et al., 2003; Yu et al., 2004). *AGL24* functions as a flowering activator and promotes inflorescence identity, whereas *SVP* acts as a flowering repressor (Yu et al., 2002). The two genes are expressed in vegetative tissues before floral transition. The expression of *AGL24* is gradually upregulated in the inflorescence apex during floral transition and is induced by multiple signals such as autonomous, vernalization, and photoperiod pathways (Michaels et al., 2003). *SOC1*, another MADS-box genes, is upregulated in the shoot meristem during the floral transition (Liu et al., 2008) and the expression pattern of this MADS-box gene overlaps with *AGL24* (Yu et al., 2002; Michaels et al., 2003). Interestingly, AGL24 directly activates the transcription of SOC1 (Lee et al., 2008) and together these MADS domain proteins regulate *LFY* (Lee et al., 2008; Liu et al., 2008), linking floral induction with flower development. AGL24 also participates in high-order MADS-box complexes with APETELA1 (AP1) and SEPALLATA3 (SEP3), which regulates flower development (Fujita et al., 2003; de Folter et al., 2005; Gregis et al., 2008).

*SOC1* is one of the flowering pathway integrators; it regulates the expression of *LFY*, which links floral induction and floral development (Liu et al., 2008). There is genetic interaction between SOC1 and AGL24, and SOC1–AGL24 interaction has been confirmed in previous studies (Yu et al., 2002; Michaels et al., 2003). As *AGL24* is activated in one shoot apex, it promotes target gene *SOC1*. Subsequently, both genes combine together and form the AGL24–SOC1 dimer. The dimer activates directly the floral meristem identity gene *LFY* finally (Lee et al., 2008; Liu et al., 2008). Therefore, *SOC1* and *AGL24* show largely overlapping expression in the shoot apex at the moment of floral transition (Liu et al., 2008). Previous studies indicated that AGL24 have the potential to form homo- or heterodimers and to build higher order complexes with other MADS and non-MADS proteins during flower development (Fujita et al., 2003; de Folter et al., 2005; Gregis et al., 2008). For example, AGL24 have been shown to interact with AP1 and SEP3 (de Folter et al., 2005; Gregis et al., 2008). Interestingly, AGL24 was also shown to interact directly with the kinase domain of the *Arabidopsis* meristematic receptor-like kinase and to be phosphorylated by the kinase domain of the receptor *in vitro* (Fujita et al., 2003). These data show that AGL24 has multiple functions, regulating both the timing of floral transition and, later, a correct flower development.

*SVP* is expressed throughout the shoot apex meristem during vegetative development and exerts its function in the maintenance of vegetative shoot identity (Hartmann et al., 2000). Several homologs of *AGL24/SVP* have been characterized from various plant species and have been found to have functional diversity. For example, *RcMADS1* from *Rafflesia* promotes flowering in a dosage-dependent manner (Ramamoorthy et al., 2013). *INCOMPOSITA* controls floral transition and floral meristem identity in *Antirrhinum* (Masiero et al., 2004). Ectopic expression of citrus *PtSVP* in tobacco inhibited early transition of the coflorescence and prolonged coflorescence development (Li et al., 2010). Wu et al. (2012) suggest that the kiwifruit *SVP* genes may have distinct roles during bud dormancy and flowering (Wu et al., 2012). Overexpression of Medicago *SVP* genes causes floral defects and delayed flowering in *Arabidopsis* (Jaudal et al., 2014). This suggests that the members of *AGL24/SVP* are likely to have multiple molecular mechanisms in the regulation of floral development. Therefore, it is interesting to study *AGL24/SVP* gene functions in species that are distantly related to *Arabidopsis*, especially perennial woody plants, because these genes may play an important role in some specific features of woody plants such as long juvenile phase and seasonal flowering.

In this study, we report the isolation and functional characterization of a MADS-box gene from trifoliate orange (precocious trifoliate orange, *Poncirus trifoliata* L. Raf) that is closely related to *AGL24* of *Arabidopsis* and named *PtAGL24*. Sequence alignment, expression profiling, protein interactions, and function analysis with regard to this gene were performed.

## Materials and Methods

### Plant Materials and Growth Conditions

All plant materials were grown in the experimental fields of the National Citrus Breeding Center at Huazhong Agricultural University, Wuhan, China (30°28′ N, 114°21′ E, 30 m a.s.l.). The seeds of precocious trifoliate orange were collected from the mother plants to ensure the same genetic background. Then the seeds were sown in 20-cm pots containing commercial potting medium (BeiLei, Zhenjiang, China) and perlite at a ratio of 3:1 (v/v); they were watered regularly with a nutrient solution. After 2 months, the germinated seedlings were transplanted and grown in the experimental fields under field conditions. Self-pruning is a physiologic phenomenon in citrus in which shoots cease vegetative growth by automatically withering the shoot tip (Zhang et al., 2014). Previous cytological studies revealed that the floral buds of spring shoots in precocious trifoliate orange initiated differentiation immediately after self-pruning (Li et al., 2010). Tissue was isolated from spring shoots after self-pruning, including stems, mature leaves, new terminal buds (subjacent lateral buds developed into new terminal buds when spring shoots after self-pruning), active lateral buds and roots. Meanwhile, whole fruits at 30 days after flowering and flowers at full bloom were sampled, and the mature flowers were also separated into different tissues. To analyze the expression pattern of *PtAGL24* during flower developmental stages, flower buds were collected at five stages of early floral development (before flowering), which was roughly defined by the length of flower buds as follows: stage 1: 1–2 mm (floral bud burst), stage 2: 2–3 mm, stage 3: 3–4 mm, stage 4: 4–5 mm, and stage 5: 5–6 mm. All samples were collected from three groups of trees and were used as biological repeats. All the samples were collected, immediately frozen in liquid nitrogen, and stored at -80°C until their used.

### RNA Extraction, First-Strand cDNA Synthesis and Isolation of *PtAGL24*

Total RNA was isolated using the Plant RNAiso Plus according to the manufacturer’s instructions (Takara, Kusatsu, Japan). The RNA samples were treated with 10 U DNase (Promega, Madison, WI, USA) for 30 min at 37°C and then further purified before real-time PCR. Approximately 2 μg total RNA was used as a template for first-strand cDNA synthesis by using the ReverTra Ace-α-cDNA Kit in accordance with the manufacturer’s protocols (Toyobo, Osaka, Japan). To obtain the full-length cDNA sequence of *PtAGL24*, the 5′ and 3′ rapid amplification of cDNA ends (RACE) strategies were performed by using the SMART^TM^ RACE cDNA Amplification Kit (Clontech, Mountain View, CA, USA) according to the manufacturer’s instructions. Thus, a pair of gene-specific primers AGL24-01 and AGL24-02 (Supplementary Table S1) was designed based on the untranslated regions for amplifying the full-length cDNA sequence. The purified PCR products were cloned into pMD18-T vector (Takara, Kusatsu, Japan) and at least three clones were selected for sequencing. The *PtAGL24* sequence has been deposited in GenBank under Accession no. KX066065.

### Sequence Alignment and Phylogenetic Analysis of PtAGL24

A phylogenetic tree based on predicted amino acid sequence and nucleotide sequence of the coding region of *PtAGL24* was constructed by using the neighbor-joining (NJ) method of MEGA4 software (Tamura et al., 2007). Amino acid sequence of PtAGL24 was predicted using DNAMAN software (version 4.0; Lynnon Biosoft, San Ramon, CA, USA), and multiple alignments were performed using ClustalW2 program and UNIPROT (Consortium, 2008). Bootstrap values were derived from 1000 replicate runs. The amino acid sequence of PtAGL24 was aligned with homologous protein sequences from various plants through BLASTN. All the sequences were downloaded from the NCBI database.

### Analysis of *PtAGL24* Transcript Level in Precocious Trifoliate Orange

To investigate the expression pattern of *PtAGL24*, various samples from precocious trifoliate orange were collected according to the experimental demands. For semi-quantitative PCR, first-strand cDNA was synthesized with oligo (dT) primer by using 1 μg DNase-treated total RNA according to the manufacturer’s instructions (Toyobo, Osaka, Japan). Then, the reverse-transcription product was diluted up to 60 μl with distilled water, 1 μl RT mixture was used as template in a 20-μl PCR reaction for 34–36 cycles, and β*-actin* was amplified as an internal control for 30–32 cycles. The PCR products were separated on 1.5% agarose gels and sequenced. All real-time PCR experiments were performed three times to validate each result. The expression level of *PtAGL24* was measured by real-time PCR using the SYBR Green PCR Master Mix (Roche Applied Science, Mannheim, Germany) as described previously (Zhang et al., 2011). Real-time quantitative PCR was performed in four replicates for each sample, and data were presented as mean values ± SD (*n* = 4). Three biological repeats were assayed in this study, giving similar trends. Data from one biologic repeat are presented. Primers used for the expression analysis are shown in Supplementary Table S1.

### Construction of Expression Vectors

To produce a vector for the constitutive expression of *PtAGL24*, the coding sequence of *PtAGL24* was amplified with AGL24-11 and AGL24-12 primers (Supplementary Table S1), which contained *Nco* I and *BstE* II restriction enzyme sites. The amplified PCR fragments were digested and then subcloned into the pCAMBIA1301 vector (CAMBIA, Canberra, Australia). Approximately 1.5 Kb of *PtAGL24* promoter was cloned into pCAMBIA1391Z vector (CAMBIA, Canberra, Australia) to drive the *GUS* reporter gene by using a pair of primers (AGL24P-1 and AGL24P-2; Supplementary Table S1). All resulting recombinant plasmids were sequenced to verify the absence of PCR errors.

### Subcellular Localization of PtAGL24

The open reading frame without the terminator codon of PtAGL24 was made into the pCAMBIA1302 by fusing to the green fluorescent protein (GFP) using the restriction enzyme *Nco* I. The *35S::GFP* was used as a control. These recombinant plasmids were transformed into onion epidermal cells (*Allium cepa* L.) by means of particle bombardment as previously described (Varagona et al., 1992). After 24-h incubation on MS medium under dark conditions at 25°C, nuclei were stained with 4′,6-diamidino-2-phenylindole (DAPI, Beyotime, Shanghai, China) in phosphate-buffered saline for 10 min. Then, GFP and DAPI fluorescence were monitored under a 90i Nikon fluorescence microscope (Nikon, Tokyo, Japan).

### *Arabidopsis* Transformation and Phenotypic Analysis

The wild-type *Arabidopsis* (Col-0) was used for transformation to confirm the function of *PtAGL24* by using the floral dipping method (Clough and Bent, 1998). T_0_ seeds were selected on medium containing 25 mg/l Hygromycin and grown under long-day conditions (16 h light/8 h dark) at 25°C. The transgenic plants T_1_ and T_2_ were also confirmed by PCR amplification. To investigate flowering time, day to flowering, and the number of rosette leaves of the third generation of *PtAGL24* transgenic lines (at least three lines) were counted when plants bore a 1-cm-long inflorescence. To evaluate the transgene effect of *PtAGL24*, real-time PCR was used in wild-type and transgenic *Arabidopsis*. For quantifying the expression levels of some endogenous flowering genes in transgenic lines, real-time PCR was also performed. The expression assay was performed in at least three independently transgenic plants. The flowers of *35S::PtAGL24* and wild-type were used for scanning electron microscopy analysis using the JEOL scanning electron microscope (JSM-6390LV, Japan) as described previously (Gregis et al., 2008). The data were processed using one-way analysis of variance (ANOVA), and statistical differences were compared based on Student’s *t*-test, with taking *P* < 0.05 considered significant.

### Yeast Two-Hybrid Assay

The experimental procedures of yeast two-hybrid assay were performed using the Matchmaker Two-Hybrid System (Clontech). The full coding sequence of *PtAGL24* was cloned into pGBKT7 vector, resulting in BD–PtAGL24 fusion protein. To test the possible interactions of PtAGL24 protein with SOC1 and AP1 clade proteins, the open reading frames of *PtSOC1* and *PtAP1* were cloned into pGADT7 using gene-specific primers (Supplementary Table S1). In addition, the open reading frame of *PtAGL24* and *Arabidopsis AGL24* (NM_118587.5), *SVP* (NM_127820.3), *SOC1* (NM_130128.3), *SEP3* (NM_102272.3), and *FLC* (NM_001161231.2) were amplified for yeast two-hybrid assay (Supplementary Table S1) and cloned into pGADT7 and pGBKT7 vectors, respectively. The truncated version of *AP1* (Z16421.1) without the trans-activating C-terminus was tested in the assay. Potential interactions were assayed on selective SD/-Trp/-Leu/-His/-Ade/X-α-gal (40 μg/ml) media supplemented with 5 mM 3-amino-triazole (3-AT).

## Results

### Amino Acid Comparison and Phylogenetic Analysis of PtAGL24

**FIGURE 1:**
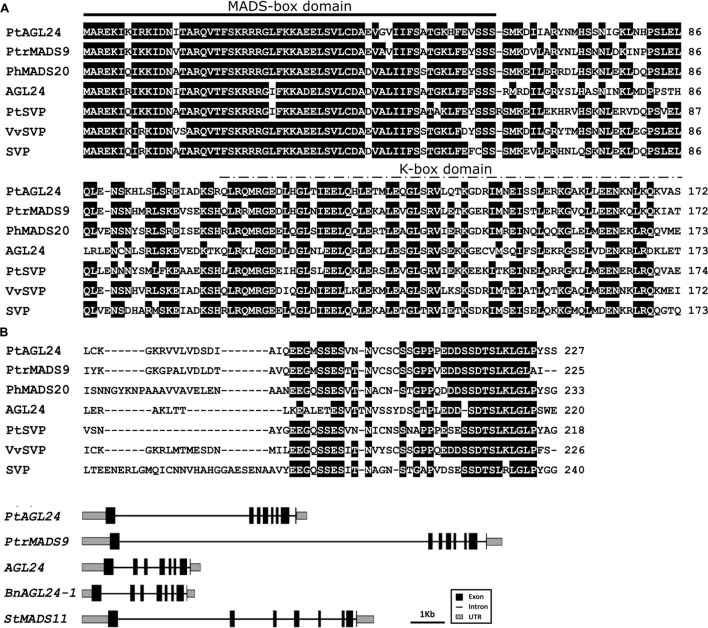
**Sequence alignment and structure analysis of *PtAGL24*.** **(A)** Comparison of PtAGL24 protein with related STMADS subfamily proteins from *Populus trichocarpa* (PtrMADS9: XM_002301057), *Petunia hybrida* (PhMADS20: GU129907.1), *Arabidopsis* (AGL24: NM_118587.5; SVP: NM_127820.3), *Poncirus trifoliata* (PtSVP, FJ373210.1), and *Vitis vinifera* (VvSVP, XM_002285651.2). Identical amino acids are shaded in black. The heavy black line indicates conserved MADS-box and the dashed line represents K-box. **(B)** Schematic representation of gene structure of *PtAGL24* and its putative homolog in *Populus trichocarpa* (*PtrMADS9*), *Arabidopsis* (*AGL24*), *Brassica napus* (*BnAGL24*), and *Solanum tuberosum* (*SVP/AGL24*).

The full length of *PtAGL24* was isolated from precocious trifoliate orange by the RACE method. The open reading frame of *PtAGL24* consists of 684 bp, encoding a 227 amino-acid sequence. Similar to other known MADS-box proteins, PtAGL24 also has a highly conserved MADS-MEF2-like domain at N terminus and a K-box domain in the middle region (**Figure 1A**). *PtAGL24* contains eight exons and seven introns (**Figure 1B**). Comparison with other MADS-box proteins included in databases showed that the deduced PtAGL24 had the highest similarity (79% identity) to PtrMADS9 of *Populus trichocarpa* over the entire coding region, and it also shared 60% identity with AtAGL24 (**Figure 1A**). The MADS-box region of PtAGL24 had 92% and 85% identity similarity to those of PtrMADS9 and STMADS16, respectively.

**FIGURE 2:**
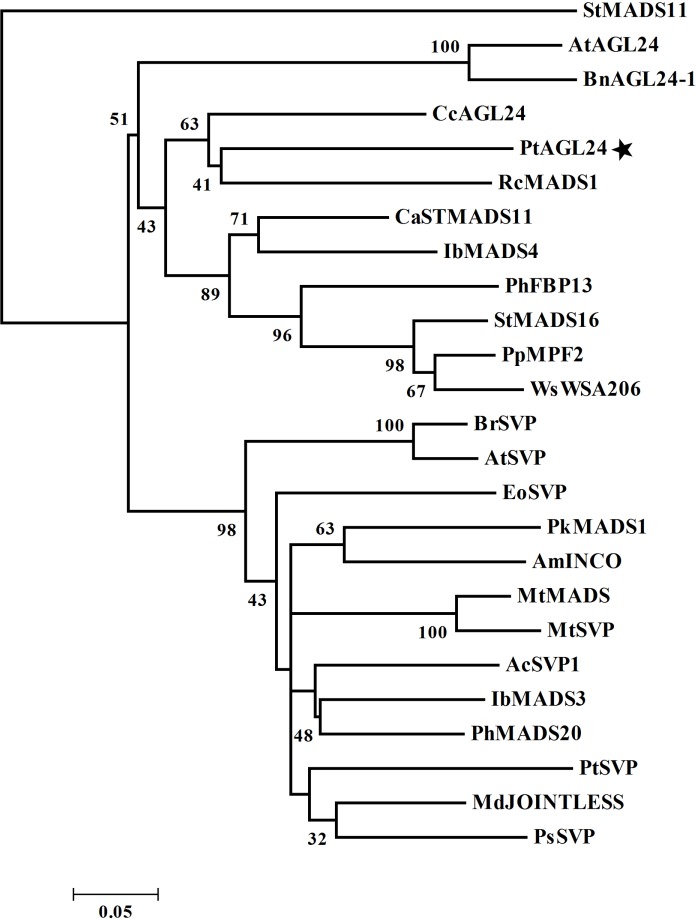
**Phylogenetic relationship of PtAGL24 and other STMADS proteins from various plants.** Bootstrap values in 1000 replicates are shown in percentages at the nodes. St, *Solanum tuberosum*; At, *Arabidopsis thaliana*; Bn, *Brassica napus*; Cc, *Carya cathayensis*; Pt, *Poncirus trifoliata*; Rc, *Rafflesia cantleyi*; Ca, *Coffea arabica*; Ib, *Ipomoea batatas*; Ph, *Petunia hybrida*; Pp, *Physalis pubescens*; Ws, *Withania somnifera*; Br, *Brassica rapa*; Eo, *Eucalyptus occidentalis*; Pk, *Paulownia kawakamii*; Am, *Antirrhinum majus*; Mt, *Medicago truncatula*; Ac, *Actinidia chinensis*; Md, *Malus domestica*; and Ps, *Paeonia suffruticosa*.

Further evidence of possible evolutionary association was seen when reported SVP/AGL24-like proteins from other plant species were considered (**Figure 2**). The evolutionary relationship between PtAGL24 and other SVP/AGL24 from various plant species were deduced using a phylogenetic analysis. PtAGL24 seem to be most closely related to RcMADS1 from *Rafflesia cantleyi* (**Figure 2**). Similar to *AGL24*, *RcMADS1* could rescue the late flowering phenotypes of *agl24-1* as ectopic expression of *RcMADS1* in *Arabidopsis* caused early flowering and conversion of sepals and petals into leaf-like structures and carpels into inflorescences (Ramamoorthy et al., 2013). These results further support that *PtAGL24* may be a homolog of *AGL24* in citrus.

### Subcellular Localization of the PtAGL24

**FIGURE 3:**
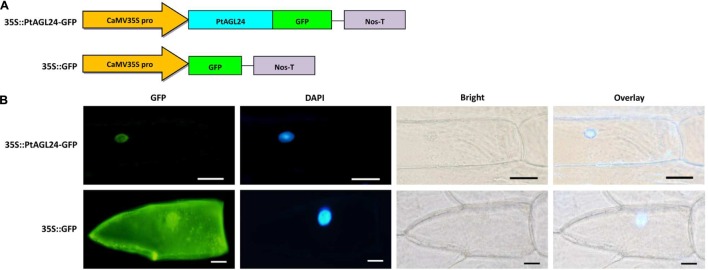
**Subcellular localization of PtAGL24 protein.** **(A)** Schematic representation of 35S::PtAGL24-GFP fusion construct and 35S::GFP construct; **(B)** subcellular localization of PtAGL24 protein in onion epidermal cells; the fluorescence signals were examined by a confocal microscopy. Nuclei of the onion cells were stained with DAPI; overlay: merged DAPI and bright-field images (scale bars: 50μm).

Previous studies have shown that AGL24 is localized to the nucleus in *Arabidopsis* (Fujita et al., 2003; Gregis et al., 2008; Lee et al., 2008). Furthermore, the predicted amino acid sequence of PtAGL24 was used for subcellular localization analysis by using PSORT prediction (Horton et al., 2007). The results indicated that PtAGL24 might be also located in the cell nucleus (data not shown). Therefore, to further determine the subcellular localization of PtAGL24, the coding sequence of PtAGL24 was fused with GFP under the control of the CaMV35S promoter (**Figure 3A**). A transient expression assay was performed in onion epidermal cells (*Allium cepa* L.). The results revealed that the 35S: PtAGL24-GFP fusion protein was mainly localized in the nucleus (**Figure 3B**). In contrast, GFP signals were observed throughout the cytoplasm and nucleus in the cells with the empty 35S:GFP control (**Figure 3B**). AGL24 localizes to the nucleus indicating that this MADS-box proteins functions as a transcription factor.

### Expression Analysis of *PtAGL24* in Precocious Trifoliate Orange

**FIGURE 4:**
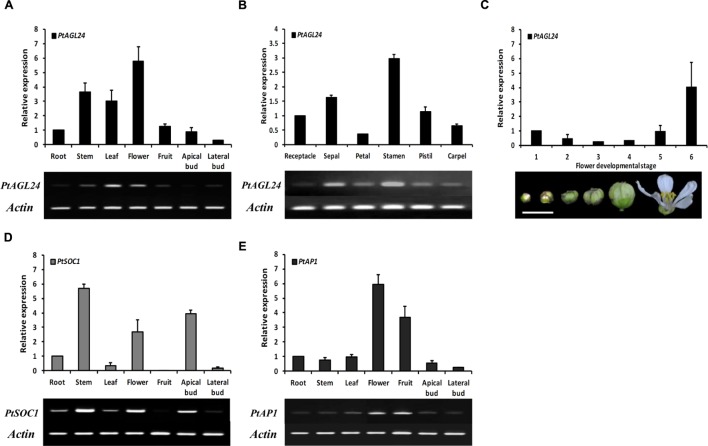
** The expression pattern of the *PtAGL24* gene in precocious trifoliate orange. The fruit is whole fruit at 30 days after flowering.** **(A,B)** Spatial expression of *PtAGL24* in various tissues and different whorls of mature flower. **(C)** The expression profile of *PtAGL24* at different developmental stages of flower (scale bar: 1 cm). **(D)** Gene expression pattern of *PtSOC1* in different tissues. The expression results were normalized to β**-actin**. Data represent the mean ± SD of four replicate reactions for the relative expression. **(E)** Gene expression pattern of *PtAP1* in different tissues.

To gain insight into the potential role of *PtAGL24*, the spatial expression pattern of *PtAGL24* was investigated in different tissues and stages of precocious trifoliate orange. The results showed that *PtAGL24* was widely expressed in almost all of the tested tissues, with relatively higher transcript levels in the fully opened flowers, stems, and leaves than in the other tissues (**Figure 4A**). *PtAGL24* also accumulated in all floral whorls of flowers in full bloom especially in the stamen (**Figure 4B**). The expression of *PtAGL24* at different stages during flower development was also investigated; the results indicated that *PtAGL24* was moderate during the early stages (from stage 1 to stage 4) and dramatically upregulated in the fully opened flowers (**Figure 4C**). These results indicated that *PtAGL24* is involved in citrus flowering and flower development. Previous studies indicated that AGL24 can interact with the SOC1 in *Arabidopsis* (Liu et al., 2008), and *AP1* can serve as a good marker to determine whether herbaceous and woody plants are at the flowering stage (Wigge et al., 2005). Therefore, the expression pattern of *PtSOC1* and *PtAP1* were investigated in this study (**Figures 4A,E**). Compared with *PtAGL24*, *PtSOC1* was strongly expressed in stems, apical buds and fully opened flowers but was barely expressed in fruits (**Figure 4D**); *PtAP1* was detected strongly in the fruits and flowers, slightly in stems and apical buds and scarcely in lateral buds (**Figure 4E**).

### Functional Analysis of *PtAGL24* in Transgenic *Arabidopsis*

**FIGURE 5:**
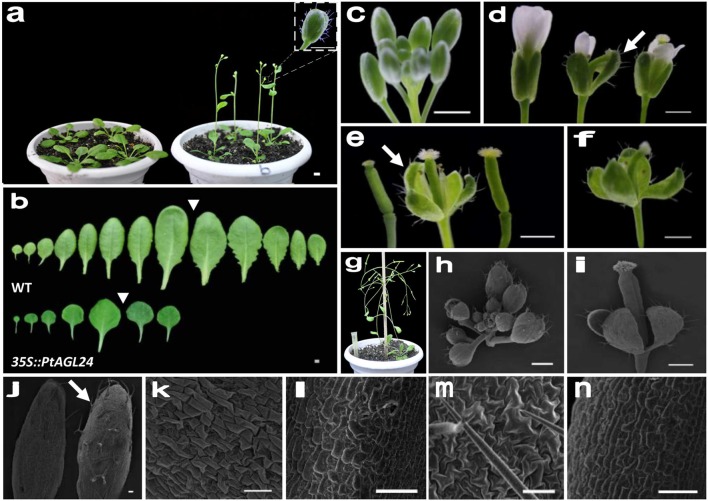
**Phenotype analysis of *PtAGL24* transgenic *Arabidopsis*.** **(a)** Accelerated flowering of class I *35S::PtAGL24* plants (right) compared with wild-type control (left). **(b)** The leaf morphologies of *35S::PtAGL24* plants before inflorescences emerged. An inverted triangle indicates the juvenile to adult transition point on the basis of the abaxial trichomes appearance. **(c)** Wild-type *Arabidopsis* inflorescence. **(d,e)** Comparison of flowers **(d)** and siliques **(e)** from wild-type (left), *35S::PtAGL24* severe phenotype with conversion of sepals into leaf-like structures (middle) and mild phenotype similar to wild-type (right). Arrows indicate leaf-like sepals. **(f)** A solitary flower of *35S::PtAGL24* after fertilization. **(g)** Mature flowers with leaf-like sepals after anthesis in transgenic plants. **(h,i)** Scanning electron microscopy (SEM) pictures of inflorescence **(h)** and mature flower **(i)** of class I *35S::PtAGL24* lines. **(j)** SEM pictures of *35S::PtAGL24* sepal (right) with enriched trichomes (arrow) compared to wild-type sepal (left). **(k–n)** SEM analysis of the cell surface morphology in wild-type sepal **(k)** and carpel **(l)** and class I *35S::PtAGL24* sepal **(m)** and carpel **(n)**, respectively. Scale bars: 1 mm **(a–g)** and 50 μm **(h–n)**.

To assess the potential roles of *PtAGL24* in the control of flowering time and the regulation of flower development, this MADS-box protein was overexpressed in *Arabidopsis*. Sixteen independent hygromycin-resistant T_1_ transgenic lines were generated and eight stable *35S::PtAGL24* transgenic lines were randomly selected and grown under long-day conditions to generate T_3_ plants for phenotypic analysis. Compared with wild-type plants (**Table 1**), all the transgenic plants showed a dramatic advance in floral transition (Student’s *t*-test, *P* > 0.05) in terms of both day to flowering and number of leaves (**Figure 5a**). The average time to flowering of the transgenic plants was about 24 days, while that of the wild-type plants was about 30 days (**Table 1**). The average number of leaves at flowering was 7 in the transgenic plants and was 12 in the wild-type plants (**Table 1**). The rosette leaves of transgenic plants were generally round and smaller than those of wild-type plants (**Figure 5b**). Based on the phenotypic variation of floral structure, the transgenic lines were classified into two groups: class I and class II. Compared with wild-type (**Figures 5c,d**), the class I flowers exhibited a severe phenotype and sepals developed into leaf-like structures with a high density of trichomes (**Figures 5d–f**). Furthermore, these leaf-like sepals subtending the flower did not detach from the developing silique after fertilization (**Figures 5f,g**). No difference in the appearance of flowers and inflorescences was observed among class II and wild-type plants except a high density of trichomes on sepals (**Figure 5d**), it is noteworthy that the density of trichomes was low compared with class I plants.

**Table 1:** Phenotypes of *35S::PtAGL24* in *Arabidopsis* under long-day conditions.

Genotype	Plants	Day to flowering^a^	Rosette leaves^b^	Note
Wild-type (*Col*)	11	30.27 ± 1.10^c^	12.18 ± 1.17	
*35S::PtAGL24*				
Class I	55	24.18 ± 1.22	7.06 ± 0.83	Leaf-like sepal
Class II	27	24.72 ± 0.92	7.12 ± 0.78	


*^a^Day to flowering is defined as the days when the inflorescence extends approximately 1 cm; ^b^Rosette leaves were counted on the day that the inflorescence extends approximately 1 cm; ^c^The average and standard error.*

**FIGURE 6:**
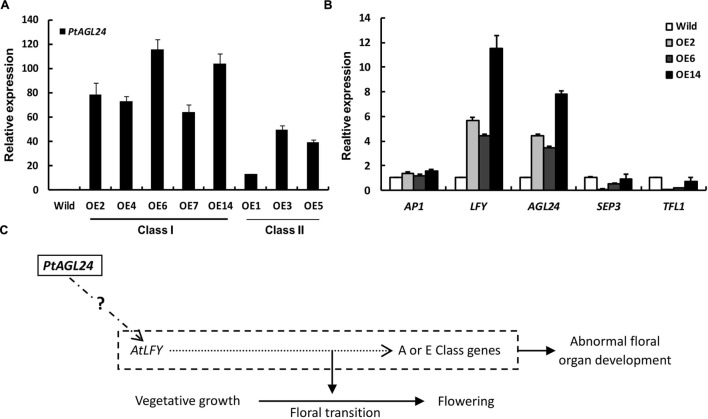
**Expression analysis of *PtAGL24* and endogenous flowering regulators in wild-type and *35S::PtAGL24* transgenic *Arabidopsis*.** **(A)** *PtAGL24* transcript levels in class I and class II transgenic lines by real-time PCR. **(B)** Expression patterns of endogenous flowering regulators in wild-type and class I. *AP1*, *LFY*, *AGL24*, *SEP3*, and *TFL1* from *Arabidopsis* were used in the analysis. The data were normalized against the expression of β*-actin*. Error bars indicate standard deviation. **(C)** A schematic representation of the involvement of *PtAGL24* in flowering regulation.

To examine the class I leaf-like sepals in more detail, the flowers of *35S::PtAGL24* and wild-type were used for SEM analysis. The results also confirmed that the transgenic plants developed aberrant floral organs with trichome-enriched sepals (**Figures 5h–j**). The sepal cells from transgenic flowers do not develop into regularly shaped cells; instead, they exhibit the sinuous and wavy epidermal cell patterning compared with wild-type plants (**Figures 5k,l**). Furthermore, the carpel epidermis of the transgenic lines distinguishes it from wild-type (**Figure 5m**), cells on the carpel surface of transgenic plants show a more dense arrangement (**Figure 5n**). To evaluate the possible relation between the expression of *PtAGL24* and abnormal phenotype of transgenic *Arabidopsis*, the expression levels of *PtAGL24* were investigated. It was revealed that the expression of *PtAGL24* was evidently high in class I, which exhibited the severe phenotype (**Figure 6A**). In addition, the expression of some endogenous flowering-related genes from *Arabidopsis* was also assessed. The levels of *AtLFY* and *AtAGL24* transcripts were clearly elevated in class I plants and proportionally lower in class II plants (**Figure 6B**). In contrast, *TFL1* and *SEP3* showed decreased expression in the transgenic lines. The expression of *AtAP1*, which has a dual role in establishing the identity of floral organs and meristems, showed little alteration (**Figure 6B**). These data suggest that *PtAGL24* functions may act as a floral activator and might be involved in citrus flowering.

### PtAGL24 Interacts with Other MADS-Box Proteins from *Arabidopsis* and Citrus

To investigate whether the phenotypic variation of floral structure in *35S::PtAGL24* plants might have been caused by PtAGL24 interact with *Arabidopsis* endogenous MADS-box proteins, the coding sequences of AGL24, AP1, SEP3, SVP, SOC1, and FLC from *Arabidopsis* were fused to the BD and AD domains and tested for their ability to interact with PtAGL24 (**Table 2**, **Supplementary Figure S1A**). The interaction analysis showed that PtAGL24 can interact with almost all the putative *Arabidopsis* AGL24 partners (AP1, SOC1 and SEP3) except FLC (**Table 2**), suggesting that the domains in the citrus and *Arabidopsis* SVP/AGL24-type protein that are important for the interactions have been conserved during evolution. Interestingly, PtAGL24 could directly interact with AGL24. This conservation of interactions between orthologs MADS-box proteins has been also observed in *Arabidopsis*, Petunia, and rice (Favaro et al., 2002, 2003; Fornara et al., 2008). In addition, the interaction among AGL24, AP1, SVP, and SOC1, which are known to interact with each other in *Arabidopsis*, was also confirmed as a control. Therefore, the interaction suggested that excessive AGL24 might cause inappropriate interactions among these transcription factors and might result in several morphological changes in transgenic *Arabidopsis*.

**Table 2:** Interactions between PtAGL24 and the *Arabidopsis* and citrus MADS-box proteins.

	AGL24		SVP		PtAGL24	
			
	AD^∗^	BD^∗∗^	AD	BD	AD	BD
AP1	+/-	+	+	+	+/-	+
SOC1	+	++		++	+	++
AGL24	+	+	–	+/-		+
SEP3		+		+		+
FLC	–	–		+	–	–
PtAP1					+	+
PtSOC1					+	+


*The number clones from cotransformation indicated the intensity of protein interaction; –, no interaction; +/-, weak interaction; +, strong interaction; ++, very strong interaction.*

*^∗^AD indicates that AGL24 was cloned into pGBKT7 and interacting protein was cloned into pGADT7.*

*^∗∗^BD indicates that AGL24 was cloned into pGADT7 and interacting protein was cloned into pGBKT7.*

Since the genetic interaction between SOC1 and AGL24 and the SOC1–AGL24 protein interaction have been reported in *Arabidopsis* (Lee et al., 2008), AGL24 has also been shown to interact with AP1 (de Folter et al., 2005). Therefore, we also performed the yeast two-hybrid assay using PtSOC1 and PtAP1 from precocious trifoliate orange to obtain insight into the functional similarity of AGL24 between citrus and *Arabidopsis* (**Table 2**, **Supplementary Figure S1B**). The results of this analysis indicate that PtAGL24 can interact with both PtAP1 and PtSOC1 (**Table 2**). This suggested that the protein–protein interaction domains of AGL24 and the formation of specific interactions with related partners might be conserved during evolution among different species.

## Discussion

Precise control of floral transition is an essential process that determines the reproductive success of flowering plants. The genetic control of flowering time and identification of flowering-related genes may have significant importance for shortening the juvenile phase and for improving citrus fruit. A subset of MADS-box proteins are involved in regulating various aspects of plant floral development (Shore and Sharrocks, 1995; Theissen et al., 2000; Jack, 2001; Becker and Theissen, 2003). In the current report, an *AGL24* homologous MADS-box gene (*PtAGL24*) was isolated from precocious trifoliate orange. Sequence alignment of the deduced amino acid sequence with other homologous revealed that PtAGL24 contains a strongly conserved MEF2-like MADS domain and a moderately conserved K-box region of the SVP/AGL24 subfamily (**Figure 1**). It shared 60% identity with AGL24 from *Arabidopsis* over the entire coding region. In agreement with this, phylogenic analysis of PtAGL24 also showed that it falls into the clade containing STMADS16, AGL24, and SVP, and it might be closer to AGL24 than to SVP (**Figure 1**). These data suggest that *PtAGL24* may be a putative *AGL24* homolog in citrus and may perform functions similar to those performed by *AGL24* in other species.

It is well known that the gene expression patterns are closely related to its functions (Chen et al., 2002). Therefore, the expression profile of *PtAGL24* was analyzed in different tissues (**Figure 4**). The results revealed that *PtAGL24* has a broad expression pattern throughout various tissues of adult plants (**Figure 4**). The accumulation of *PtAGL24* was higher in fully opened flowers, stems, and leaves, consistent with previous reports on the *AGL24* in *Arabidopsis* (Michaels et al., 2003). In *Arabidopsis*, AGL24 and SOC1 function together to regulate the floral transition and inflorescence meristem identity (Lee et al., 2008; Liu et al., 2008), while association of AGL24 with AP1 in the floral meristem regulates flower development (de Folter et al., 2005; Fornara et al., 2008). In this study, the direct interaction between PtAGL24 and PtSOC1 or PtAP1 supports this argument (**Supplementary Figure S1B**). PtAGL24 was localized in the nucleus, which is a feature of transcription factors (Fujita et al., 2003; Gregis et al., 2008). This strongly suggests that the interaction domains are conserved in these proteins and the formation of specific interactions with related partners is a conserved evolutionary feature.

Because the genetic transformation of citrus usually has low efficiency and requires a long period of time, a function analysis of *PtAGL24* was undertaken for *Arabidopsis* (**Figure 5**). The results showed that overexpressing *PtAGL24* flowered earlier than the control in transgenic *Arabidopsis*. Flowering time, in terms of number of day to flowering and number of leaves at flowering, differed significantly (*P* < 0.05) between the transgenic lines and controls under long-day conditions (**Table 1**). These results suggested that *PtAGL24* acts as a floral inducer in citrus. This is distinct from its homolog *PtSVP*, which is characterized by its maintained juvenile character and delayed flowering (Li et al., 2010). In addition, accumulating data suggest that ectopic expression of *PtAGL24* resulted in altered flower morphology phenotype similar to that of *35S::AGL24* (Michaels et al., 2003). Similarly, ectopic expression of an *AGL24* ortholog (*RcMADS1*) from the *Rafflesia cantleyi* caused early flowering and conversion of sepals and petals into leaf-like structures and of carpels into inflorescences in *Arabidopsis* (Ramamoorthy et al., 2013). In rice, ectopic expression of the *SVP/AGL24* ortholog (*OsMADS22* and *OsMADS47*) in *Arabidopsis* revealed alterations in flower development, while the flowering time phenotypes of *svp* and *agl24* mutants were not complemented (Favaro et al., 2002). These findings suggest that *PtAGL24* is involved in flowering time regulation and may influence flower development, and that the function and expression patterns of *PtAGL24* are conserved between *Arabidopsis* and citrus.

So far, MADS-box genes in the SVP/AGL24 subfamily have been isolated from various plants, and overexpression of the members of this clade causes alterations in flowering time and floral morphology (Fornara et al., 2008; Ramamoorthy et al., 2013). In *Arabidopsis*, *AGL24* acts as an important integrator of multiple flowering signals and regulates flowering in a dosage-dependent manner (Yu et al., 2002). For example, the interaction of *SOC1* and *AGL24* is required for activation of *LFY* (Lee et al., 2008) consistent with our results, *LFY* expression should be increased in plants with over-expression of *AGL24*. The increased expression of *LFY*, which is directly bound and induced by *AGL24*, is central to the transition to flowering at the site of floral meristem formation in *Arabidopsis* (Lee et al., 2008). *TFL1* is a key gene for maintenance of the inflorescence meristem by preventing the expression of floral meristem identity genes such as *AP1* and *LFY* in the central dome of the shoot apical meristem (Wu et al., 2012; Jaudal et al., 2014). *SEP3* is important for determining floral organ identity (Pelaz et al., 2000); the single *sep3* mutants displayed partial transformation of the petals into sepals (Pelaz et al., 2000; Wang J.-W. et al., 2009). *Arabidopsis* endogenous *SEP3* and *TFL1* expression were strongly repressed in transgenic *Arabidopsis* in this study. These results might correlate with early flowering and changed morphological phenotype. In contrast, the expression of *AP1*, which is principally required to direct the development of floral organ (Bowman et al., 1993), did not show significant alterations in these transgenic lines. Interestingly, endogenous *AGL24* expression was also strongly increased in transgenic *Arabidopsis*. The yeast two-hybrid assay showed that PtAGL24 interacts with AP1, AGL24, and other partners of AGL24; PtAGL24 may need more AGL24 interaction in transgenic *Arabidopsis.* This suggests that the interaction domains of these proteins are conserved, and the interaction between exogenous and endogenous proteins in an inappropriate moment of floral development might be attributed to the alteration of floral morphology (**Figure 6c**). Taken together, the results indicate that *PtAGL24* is a functional ortholog of *Arabidopsis AGL24*, and it may be recruited as a critical integrator of flowering inducers in flowering time control and plant architecture in citrus.

In summary, we demonstrated that *PtAGL24* acts as a transcription factor correlated with the floral transition by transgenic *Arabidopsis* expressing *PtAGL24* and may be involved in meristem maintenance in citrus. Although the rest of the regulation mechanisms of the process are not understood at this time, our study suggests that the function of *PtAGL24* in citrus may be well conserved. Therefore, further efforts will be made to find more direct evidence, including complementing the *agl24 Arabidopsis* mutant and ectopic expression of *PtAGL24* by transformation in citrus. In addition, further studies are required to understand how *PtAGL24* is regulated or how it regulates other genes involved in flowering in citrus, and whether it directly regulates the flowering promoter SOC1 to accelerate the floral transition. Answers to these questions will greatly improve our understanding of the annual flowering mechanisms of citrus and other woody plants.

## Author Contributions

J-ZZ and L-MS wrote the paper. J-ZZ and C-GH participated in research design. C-GH and J-ZZ were responsible for generating the data and for interpreting the results.

## Conflict of Interest Statement

The authors declare that the research was conducted in the absence of any commercial or financial relationships that could be construed as a potential conflict of interest.

## Supplementary Material

**FIGURE S1 | PtAGL24 interacts with other MADS-box proteins from *Arabidopsis* and citrus by yeast two-hybrid analysis.** **(A)** PtAGL24 interacted with *Arabidopsis* AtAGL24 and other partners of AtAGL24 by a yeast two-hybrid assay; 1: BD-p53/AD-RceT; 2: BD-PtAGL24/AD-AtAGL24; 3: BD-PtAGL24/AD-AtAP1; 4: BD-PtAGL24/AD-AtSEP3;5: BD-PtAGL24/AD-AtSOC1. **(B)** PtAGL24 interacted with citrus PtSOC1 and PtAP1.
