# Supplementary figures and images for: Characterization and Expression Analysis of PtAGL24, a SHORT VEGETATIVE PHASE/AGAMOUS-LIKE 24 (SVP/AGL24)-Type MADS-Box Gene from Trifoliate Orange (Poncirus trifoliata L. Raf.)
Source: Front Plant Sci. 2016 Jun 10;7:823. doi: 10.3389/fpls.2016.00823 (PMC4901042; doi:10.3389/fpls.2016.00823)

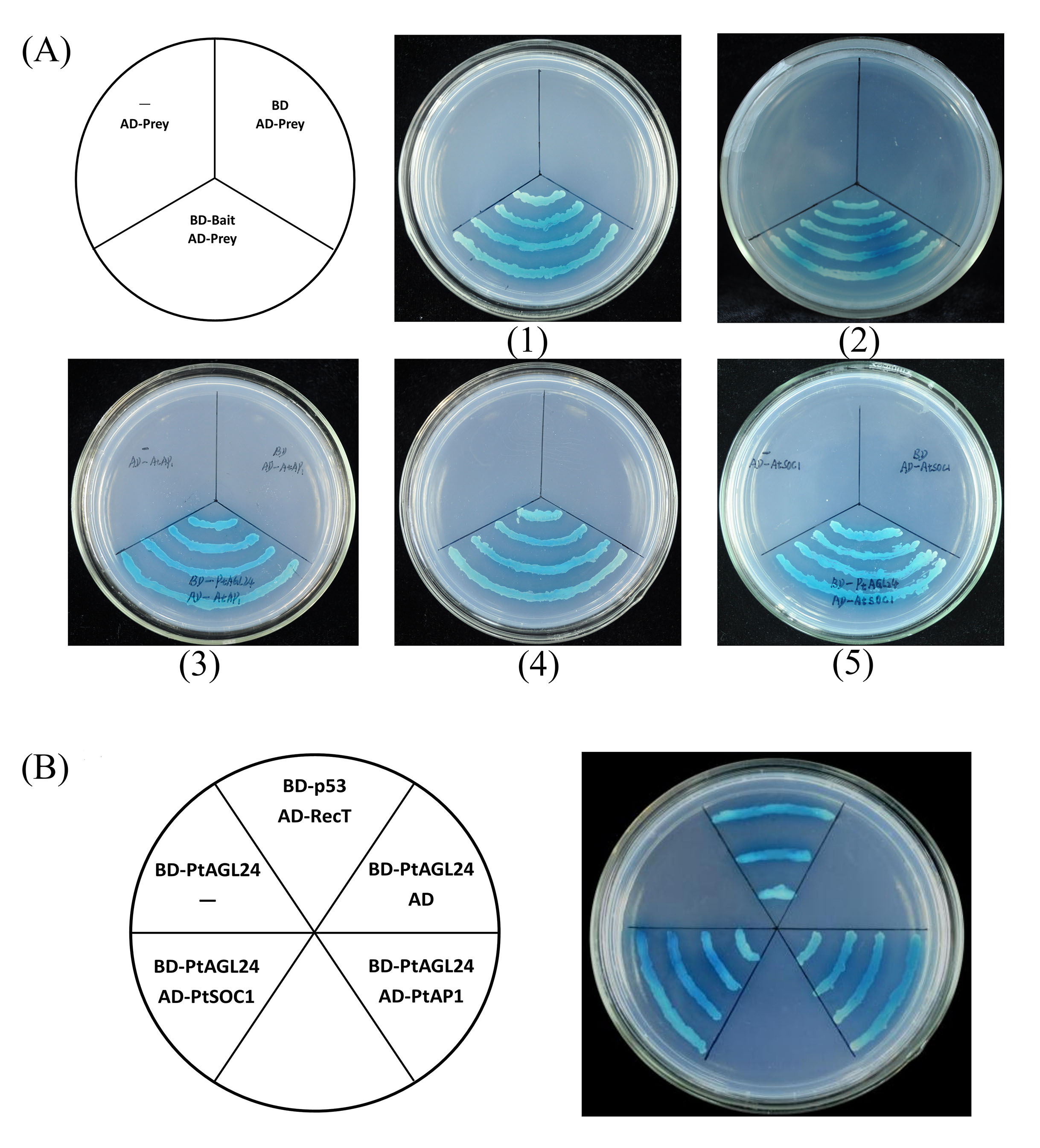

Supplement: Supplementary file 1 [file Image_1.JPEG]
