# Supplementary material for: Characterization and Expression Analysis of PtAGL24, a SHORT VEGETATIVE PHASE/AGAMOUS-LIKE 24 (SVP/AGL24)-Type MADS-Box Gene from Trifoliate Orange (Poncirus trifoliata L. Raf.)
Source: Front Plant Sci. 2016 Jun 10;7:823. doi: 10.3389/fpls.2016.00823 (PMC4901042; doi:10.3389/fpls.2016.00823)
Supplement: Supplementary file 2 [file Table_1.PDF]

Supplementary Table S1. The primer sequence information in this study

The primer for gene amplication:		
PtAGL24	Forward(AGL24-01):	5`-TTGGTGGCTTTAGGTTACTG-3`
	Reverse(AGL24-02):	5`-TCCATAAACACAACAGCATCT-3`
Two pairs of primers for walking:		
PtAGL24	GSG01:	5`-TCGCTGTTATGTTATCAATCTTC-3`
	GSG02:	5`-CAAAATGGCGATAATTCATAAT-3`
	GSG03:	5`-TGGAGTCCTTCTGAGGTGCGTC-3`
	GSG04:	5`- ATTTTTTCTAATACTGGGTGG-3`
The primers for vector construct:		
PtAGL24	Forward(AGL24-11):	5`-CCATGGGGCTTTAGGTTACTGTGATC-3
	Reverse(AGL24-12):	5`-GGGTAACCTCCATAAACACAACAGCATCT-3`
PtAGL24P	Forward(AGL24P-1):	5`- GGATCCGATAAGATAAATCGGAAAT-3`
	Reverse(AGL24P-2):	5`- GAATTCAATAATATATAATAAGCAGA-3`
The primers for semi-quantitatively RT-PCR:		
PtAGL24	Forward:	5`-TTCTTTGTGATGCTGAGGTTGG-3`
	Reverse:	5`-GATGACATGCCTTCTTCTTGGA-3`
PtSOC1	Forward:	5`-TTCTCTGTGATGCTGAGGTTG-3`
	Reverse:	5`-TCGTCGTTTGTTAGATTCTCT-3`
β-Actin	Forward:	5`-CCGACCGTATGAGCAAGGAAA-3`
	Reverse:	5`-TTCCTGTGGACAATGGATGGA-3`
The primers for Real-time quantitatively RT-PCR:		
PtAGL24	Forward:	5`-TGTGATGCTGAGGTTGGTGTC-3`
	Reverse:	5`-GGATGATTCAATTTCCCGATG-3`
PtSOC1	Forward:	5`-GAAACAAGCAACAGCCAACG-3`
	Reverse:	5`-AAGGGTGCATGATGCCAGAC-3`
AtAP1	Forward:	5`-CATGGGTGGTCTGTATCAAGAA-3`
	Reverse:	5`-CATGCGGCGAAGCAGCCAAGGTT-3`
AtLFY	Forward:	5`-AGAGGGAGCATCCGTTTATC-3`
	Reverse:	5`-TCGCCACGGTCTTTAGCAAT-3`
AtAGL24	Forward:	5`-ACGCTTAAAGAGGCTTTGGA-3`
	Reverse:	5`-CAGGGAAGTGTCGGAGTCAT-3`
AtSEP3	Forward:	5`-CGGTCGTCATCATCATCAACA-3`
	Reverse:	5`-GTCCTGCTCCCATTCCATCTT-3`
AtTFL1	Forward:	5`-GCCAAGCATAGGGATACATAG-3`
	Reverse:	5`-ACAGGGAGACCAAGATCATAC-3`
β-Actin	Forward:	5`-CCGACCGTATGAGCAAGGAAA-3`
	Reverse:	5`-TTCCTGTGGACAATGGATGGA-3`

The primers for yeast two-hybrid assay:		
PtAGL24	Forward:	5`-GAGAAGATCAAGATCAGGAA-3`
	Reverse:	5`-AGTTCAGCTGGAGTAGGGAA-3`
PtSOC1	Forward:	5`-GTGAGAGGCAAAACTCAA-3`
	Reverse:	5`TGATCAGGTTTGTGGTGGTATTG-3`
PtAP1	Forward:	5`-GGAAGAGGTAGGGTTCAGCTGAAG-3`
	Reverse:	5`-TCTCACAGTTCATCCAGCAAAGCA-3`
AtAGL24	Forward:	5`-GCGAGAGAGAAGATAAGGAT-3`
	Reverse:	5`-GATTCATTCCCAAGATGGAAG-3`
AtAP1	Forward:	5`-GGAAGGGGTAGGGTTCAATT-3`
	Reverse:	5`-TCATGCGGCGAAGCAGCCAA-3`
AtSOC1	Forward:	5`-GTGAGGGGCAAAACTCAGAT-3`
	Reverse:	5`-TCACTTTCTTGAAGAACAA-3`
AtSEP3	Forward:	5`-GGAAGAGGGAGAGTAGAATT-3`
	Reverse:	5`-TCAAATAGAGTTGGTGTCAT-3`
AtFLC	Forward:	5`-GGAAGAAAAAAACTAGAAATC-3`
	Reverse:	5`-CTAATTAAGTAGTGGGAGAG-3`
AtSVP	Forward:	5`-GCGAGAGAAAAGATTCAGAT-3`
	Reverse:	5`-CTAACCACCATACGGTAAGC-3`
